# 胸腔积液沉淀物在恶性胸腔积液诊断中的临床应用价值

**DOI:** 10.3779/j.issn.1009-3419.2017.06.05

**Published:** 2017-06-20

**Authors:** 欣同 王, 方圆 程, 殿胜 钟, 丽沙 张, 凡路 孟, 宜 邵, 涛 于

**Affiliations:** 300052 天津，天津医科大学总医院肿瘤科 Department of Medical Oncology, Tianjin Medical University General Hospital, Tianjin 300052, China

**Keywords:** 恶性胸腔积液, 自然静止法, 血液凝集法, 免疫组化染色, 临床价值, Malignant pleural effusion, Natural sedimentation, Plasma coagulation method, Imunohistochemical staining, Clinical value

## Abstract

**背景与目的:**

恶性胸腔积液（malignant pleural effusion, MPE）是原发于胸膜或转移至胸膜的恶性肿瘤造成的胸腔积液。MPE患者预后差，应在减少病人痛苦的前提下，准确而快速的明确MPE的性质及病因，为后续治疗提供有效依据。

**方法:**

103例患者应用自然静止法或血凝集法制得胸水沉淀物，结合HE染色及免疫组化染色，在诊断MPE上与其他方法相比较。

**结果:**

103例MPE中，胸腔积液沉淀物方法确诊90例（诊断率87.4%）；32例仅通过沉淀物诊断，74例指出病理类型，23例明确原发灶；与71例同时行有创方法比较，诊断率为81.7%与87.3%；对比液基细胞学，检出率为86.7%和44.0%。

**结论:**

胸腔积液沉淀物方法不仅可以增加细胞学诊断率且与其他有创方法诊断率近乎一致，同样可确定MPE病理类型及原发灶，是有创方法的较佳补充，甚至对于部分患者胸水沉淀物是唯一确诊方法。

## Abstract (en)

**Background and objective:**

Malignant pleural effusion (MPE) is due tumor which arises from the mesothelium or metastases from tumor origniating other sites. Generally, the prognosis of MPE is poor, in the premise of reducing the pain of patients, as soon as possible make clear the property of pleural effusion and cause of the disesease, rightly and quickly, providing effective information for subsequent treatment.

**Methods:**

The cell block of 103 patients by using natural sedimentation or plasma coagulation method combined with HE staining and immunohistochemical staining method maked clear diagnosis and compared with other methods.

**Results:**

90 patients were diagnosed by cell block section from 103 patients who had MPE (diagnostic rate 87.4%); 32 cases were diagnosed by cell block section only, 74 cases pointed out that the pathological type, 23 cases even pointed out the primary lesions; 71 cases examined other invasive methods at the same time, the diagnostic rate was 87.3% and 81.7%; the detection rate of cell block section and cytological smear in detecting malignant tumor cells was 86.7%and 44.0% respectively.

**Conclusion:**

Cell block can not only increase the diagnosis, in contrast to cytological smear, and own the same diagnostic rate compared with other invasive methods, but also can confirm pathological type and primary lesion; especially, for other invasive methods, cell block method is a preferable complementary method, and that cell block method maybe the only way for some patients.

恶性胸腔积液（malignant pleural effusion, MPE）由胸膜的原发恶性病变或转移或侵袭至脏壁层胸膜而引起的胸腔积液。美国每年发生恶性胸腔积液的人数超过150, 000人^[1]^。据统计，MPE最常见的病理分型是腺癌，肺癌是常见原因^[2]^。一旦确诊MPE则可能失去手术机会，且预后较差，中位生存期仅3个月-12个月^[3]^。因此，应在尽量减少患者痛苦的前提下，准确而快速的明确胸腔积液的性质或引起MPE的原因，为后续治疗提供有效的依据。胸腔镜、气管镜、胸水细胞学等^[3, 4]^是临床确诊MPE常用方法。胸腔镜因存在有创性，部分患者不能进行，且普及率不高，而细胞涂片则诊断率不高且在鉴别细胞类型上有一定的难度。胸腔积液沉淀物则可以通过结合相关化学染色，不仅将MPE诊断率提高^[5, 6]^，甚至可指出原发病灶^[7]^。作者回顾性分析2010年4月-2016年10月在我院呼吸科及肿瘤科就诊的103例恶性胸腔积液患者，结合胸腔积液沉淀物相关临床资料，探讨胸腔积液沉淀物在MPE诊断中的独特临床优势。

## 1 资料与方法

### 1.1 临床资料

收集2010年4月-2016年11月就诊于天津医科大学总医院呼吸科及肿瘤科的103例恶性胸腔积液患者，通过自然静置法或血液凝集法留取了沉淀物。其中，男性49例，女性54例，最小年龄32岁，最大年龄92岁，平均年龄67岁。有71例患者同时行手术、胸腔镜、经皮胸膜穿刺、支气管镜、肠镜、PET-CT等检查；32例患者仅通过胸水沉淀物确定MPE，甚至原发病灶。75例患者同时送检胸水液基细胞学检查。

### 1.2 方法

#### 1.2.1 液基细胞学

患者行穿刺引流术后，取胸水15 mL于试管，3, 000 r/min，离心10 min，弃上清液，取沉渣涂片3张，95%乙醇固定，HE染色，光镜观察。

#### 1.2.2 胸腔积液沉淀物

① 自然静置法留取沉淀物：患者行胸腔穿刺引流积液至引流袋中，留取600 mL-1, 000 mL，放置于无污染区静置2 h-6 h，待出现块状沉淀物后，弃上清液，用无菌镊子或无菌棉签将沉淀物取出（图 1A）。②血液凝集法制作沉淀物若引流液在静置后仍未形成满意的块状沉淀物，可采用血液凝集法制作沉淀物。步骤如下：首先将部分上清液排出，抽取底部2-5管（10 mL/管）沉淀液置离心管中，1, 000 rpm，离心5 min，弃上清液，分别将离心管底部少量沉淀物放入无菌的标本盒中，抽取患者的新鲜血液1 mL，加入标本盒中，静置半小时，形成血凝块。用无菌镊子或者是无菌棉签将血凝块取出（图 1B）。将上述方法获得的沉淀物放入10%的中性福尔马林溶液中固定，送病理科，常规脱水、石蜡包埋、切片，HE染色，免疫组化染色。

**1:**
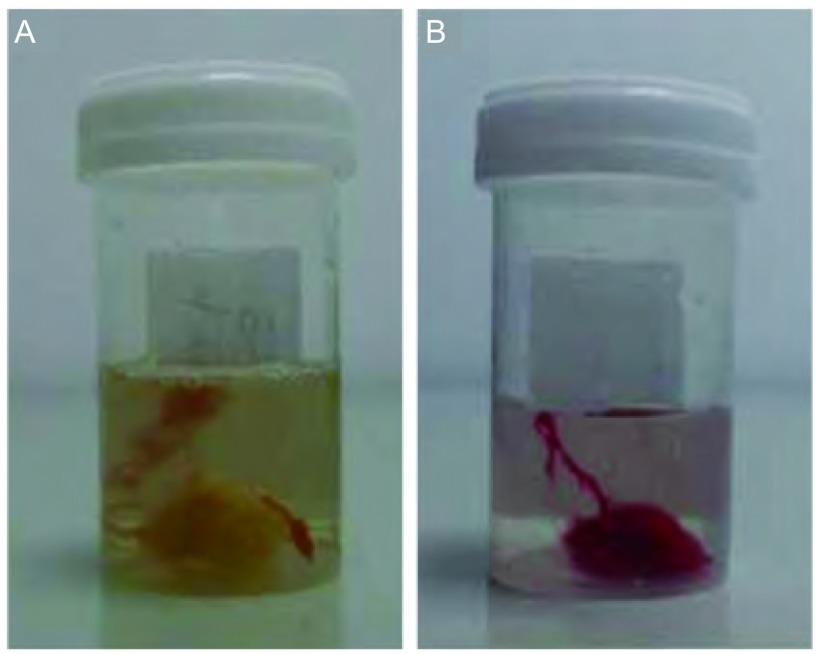
标本获取方式。A：静置法沉淀物标本；B：血凝集法沉淀物标本 Methods obtaining cell block specimens. A: the specimen of natural sedimentation; B: the specimen of plasma coagulation method

### 1.3 统计学方法

本研究相关的统计学分析采用SPSS 17.0统计软件进行统计学处理，计数资料的比较应用卡方检，以*P* < 0.05表示差异具有统计学意义。

## 2 结果

### 2.1 胸腔积液沉淀物的确诊情况

在103例恶性胸腔积液中，通过沉淀物方法确诊的有90例（诊断率87.4%）（表 1），13例阴性的患者，经气管镜等有创操作确诊恶性肿瘤病变，经临床及胸部CT及胸水常规或细胞学等相关检查证实为恶性胸腔积液。

**1:** 90例胸腔积液沉淀物明确诊断情况 90 patients diagnosed by cell block

Primary lesion	Lung	Ovary	Breast	Colon	Stomach	Pancreas	Kidney	Liver	Esophagus	Pleurope-ritoneum	Un-certain	Total
Adenocarcinoma	51	4	2	1	1	0	0	1	0	1	0	61
^*^Squamous cell	2	0	0	0	0	0	0	0	0	0	0	2
Small cell carcinoma	7	0	0	0	0	0	0	0	0	0	0	7
Mesothelioma	0	0	0	0	0	0	0	0	0	3	0	3
Sarcomatoid arcinoma	1	0	0	0	0	0	0	0	0	0	0	1
Uncertain	7	2	0	0	0	1	1	0	1	0	4	16
Total	68	6	2	1	1	1	1	1	1	4	4	90
*1 cell block diagnosed lung of adenocarcinoma, but percutaneous lung puncture biopsy diagnosed small cell carcinoma.												

#### 2.1.1 71例患者同时行气管镜等方法与胸腔积液沉淀物方法结果比较

71例患者通过两种诊断方法比较，胸水沉淀物方法明确诊断58例，诊断阳性率为81.7%；气管镜等其他方法明确诊断62例，诊断阳性率为87.3%。其中诊断一致的有47例，诊断一致率为66.2%。不一致的有24例，有9例虽做气管镜等检查但诊断未明确，有6例行气管镜结果阴性，未见肿瘤细胞，3例PET-CT虽指出多部位代谢增高，但未具体明确诊断，上述病例则通过胸水沉淀物方法明确恶性病变乃至病理类型，甚至指出原发病灶来源（表 2）。

**2:** 71例患者胸腔积液沉淀物方法与其他检查方法结果比较 Conventional cell block and other methods diagnostic results

Cell block	The other methods		Total
	Positve	Negtive or unclear	
Positive	47^#^	9^*^	56
Negtive or inconformity	15^※^	0	15
Total	62	9	71
^#^: 5 cases dignosed by cell block at first and provided information to other methods, then other methods confirmed. 21 cases dignosed by cell block and other methods at the same time. 21 cases who before dignosed by cell block had diagnosis. ^*^: Although 6 cases dignosed by bronchoscope at first, the results were negative. 3 cases examined PET-CT before cell block, but the results pointed out various lesions and cannot give a definitive diagnosis, cell block pointed out the primary lesion. ^※^: 13 cases of diagnoses were negative, 1 case of result was small cell lung cancer by percutaneous lung puncture biopsy, but cell block was adenocarcinoma, 1 case dignosed adenocarcinoma by bronchoscope, cell block dignosed large cell carcinoma at first submitted specimen, the second dignosed adenocarcinoma.			

#### 2.1.2 103例MPE患者首先确诊方法

103例患者中，首先通过胸水沉淀物方法明确诊断的有46例，占所有确诊MPE患者的44.7%。其中，32例（31.1%）患者仅通过胸水沉淀物明确诊断；5例（4.9%）通过胸水沉淀物诊断提示后，又通过其他方法证实；9例（8.7%）其他诊断方法未明确，最终由胸水沉淀物确诊（图 2）。

**2:**
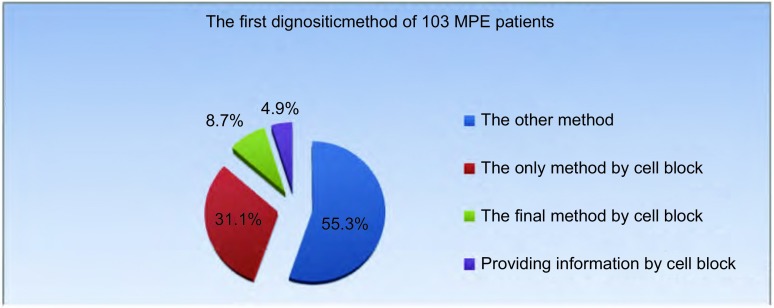
103例MPE患者首先确诊方法 The first dignositic method of 103 MPE patients

#### 2.1.3 46例胸腔积液沉淀物首先明确诊断的特殊案例

其中，6例患者首发症状仅为胸腔积液，通过胸腔积液沉淀物方法明确为恶性，有2例指出了病变来源。4例患者通过影像学检查指出两种或多种占位性病变，通过胸水沉淀物明确了原发病灶，为后续治疗指明方向（表 3）。

**3:** 典型病例摘要 Abstract of typical cases

Number	Previous history	Condition before diagnose	Result by cell block
Case 1	Breast cancer	Pleural effusion and lung space occupying lesion	Lung cancer
Case 2	Gynecologic carcinoma	Pleural effusion only	Mesothelioma
Case 3	None	Pleural effusion only	Ovarian cancer
Case 4	None	Pleural effusion, ovary and pancreatic occupying lesion	Ovarian cancer
Case 5	None	Pleural effusion, pancreatic and lung occupying lesion	Pancreatic cancer
Case 6	None	Pleural effusion, breast nodule minimal, lung occupying lesion obvious	Breast cancer

### 2.2 胸腔积液沉淀物与液基细胞学检测方法的比较

75例MPE患者同时送检胸腔积液沉淀物及液基细胞学，通过HE染色及免疫组化方法，胸腔积液沉淀物阳性率为86.7%（65例）；液基细胞学阳性率为44.0%（33例），两种方法对恶性肿瘤细胞检出率的差异有统计学意义（χ^2^=25.29，*P* < 0.005，表 4）。

**4:** 胸腔积液沉淀物及液基细胞学法对恶性肿瘤细胞检出率的比较 Conventional cytology smear and cell block technique diagnostic rations

Cell Block	Cytology Smear		Total
	Postive	Negative	
Positive	30	35	65
Negative	3	7	10
Total	33	42	75
*χ*^2^=25.29, *P* < 0.005			

### 2.3 胸腔积液沉淀物HE染色及免疫组化染色结果

总结90例由胸水沉淀物确诊的恶性病变患者，HE染色后有58例（64.4%）可见肿瘤细胞，8例（8.9%）见可疑肿瘤细胞，24例（26.7%）见核异质细胞（图 3）。其中通过结合免疫组化后，全部确诊为恶性病变。

**3:**
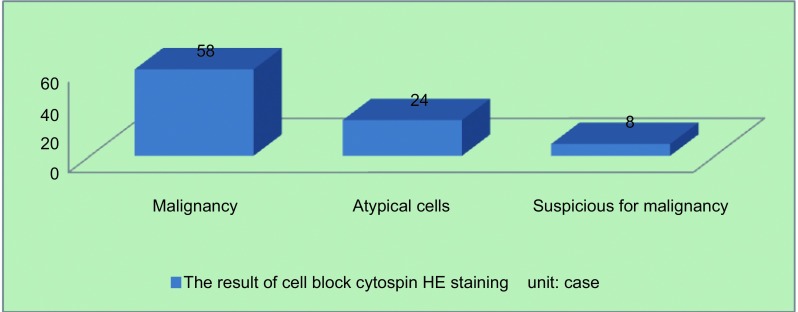
90例胸腔积液沉淀物HE染色结果 90 cases′results of cell block cytospin HE staining

90例胸腔积液沉淀物HE染色结合免疫组化染色的结果显示：腺癌（图 4A）61例（67.8%），鳞癌（图 4B）2例（2.2%），小细胞癌（图 4C）7例（7.8%），肉瘤样癌1例（1.1%），间皮瘤（图 4D）3例（3.3%），病理类型不确定16例（17.8%）。有23例（25.6%）结合免疫组化相关抗体指出原发病灶或可能来自的原发病，如来自肺的TTF-1（图 5A）和NapsinA（图 5B）。

**4:**
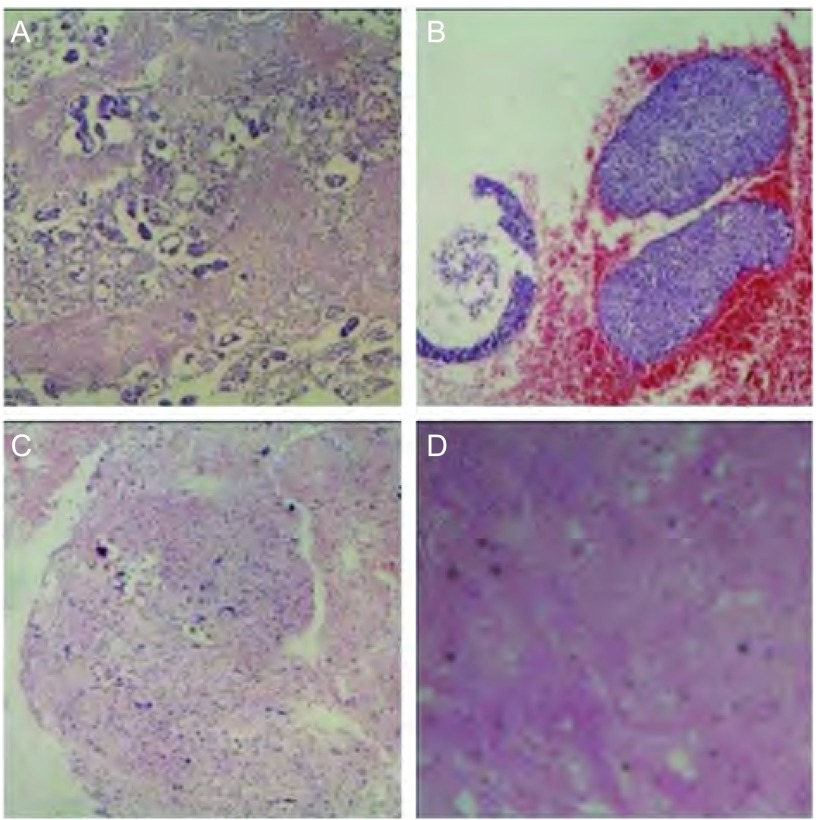
40倍显微镜下不同病理类型HE染色图片。A：腺癌；B：鳞癌；C：小细胞癌；D：间皮瘤 HE staining of dif ferent pathological t ypes (×40). A: adenocarcinoma; B: squamous cell carcinoma; C: small cell carcinoma; D: mesothelioma

**5:**
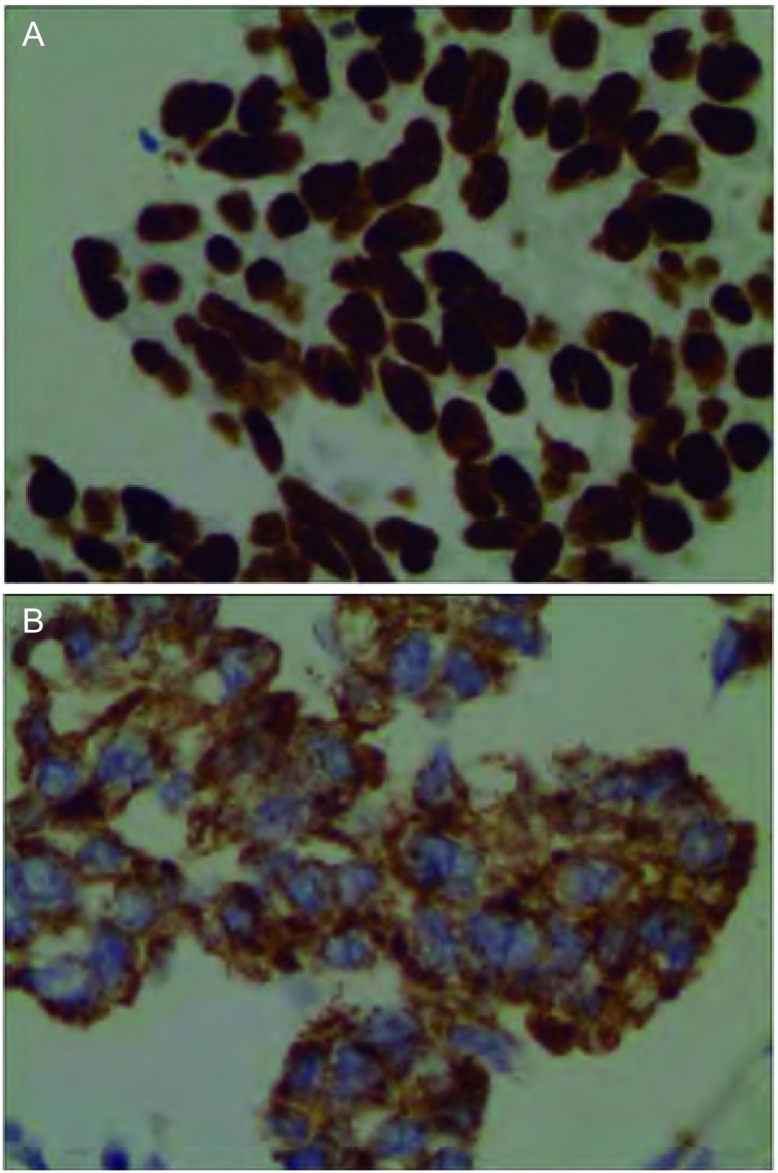
400倍显微镜下部分免疫标记物免疫组化染色图片。A：TTF-1阳性；B：Napsin A阳性 Immuno-staining of partial expression in the cells of cell block by immunohistochemistry (×400). A: TTF-1 positive; B: Napsin A positive

### 2.4 获得沉淀物标本的方式及送检标本确诊率

103例患者共送检141次沉淀物标本，其中118次（83.7%）送检标本由自然静止法获得，23次（16.3%）送检标本由血液凝集法获得。90例确诊病例中，68例（75.6%）患者由第一次送检标本确诊，19例（21.1%）患者由第二次送检标本确诊，3例（3.3%）患者送检3次及以上送检标本确诊（图 6）。

**6:**
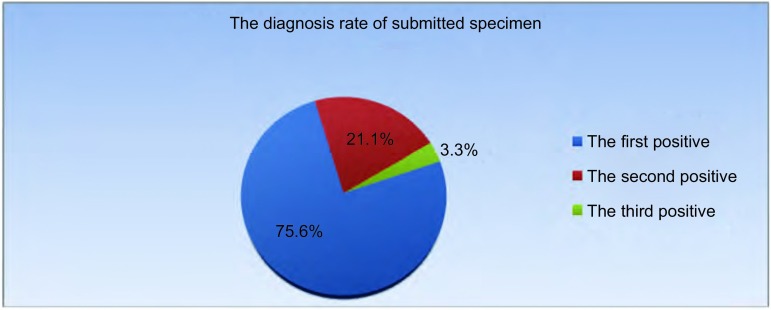
103例患者标本送检确诊率分布图 103 patients diagnosis rate of submitted specimen

## 3 讨论

几乎所有的恶性肿瘤在疾病的发生及进展的过程中均可侵袭胸膜引起MPE^[8]^。支气管镜、经皮胸膜穿刺、胸腔镜等是目前临床上常用的确诊手段。但是上述检查均有一定的有创性，除了有创性，部分患者无法进行外，胸腔镜检查即使在大医院中也不普及。胸腔积液沉淀物方法操作方便、有创性小，几乎所有的胸腔积液患者均可进行^[9]^，并且随着免疫组织化学技术的快速发展，相比细胞学方法能显著提高MPE的诊断率^[10, 11]^。李月川等^[12]^首先在文献中介绍通过自然静置法获得胸腔积液沉淀物。本研究在此基础上，对静置无法形成沉淀物的胸水，通过血凝法制作沉淀物，最大可能获取患者诊断价值的有利信息。

本研究中103例MPE患者，胸水沉淀物方法确诊率为87.4%，而且32例仅行沉淀物明确诊断。胸腔积液沉淀物检查对于原发病灶的诊断亦有很大的帮助^[13, 14]^。本研究中，46例由胸水沉淀物首先确诊的患者，既有仅表现为胸腔积液者；也有胸腔积液伴发多处占位性病变患者，需要明确原发病灶；还有气管镜等检查未能明确诊断者。这些患者通过胸水沉淀物，最终明确了胸水性质，甚至病变来源，找到了原发病灶，为后续治疗指明方向。所以，胸水沉淀物可作为其他检查的补充方法，甚至可能是唯一的确诊方法。

本研究中，胸腔积液沉淀物和液基细胞学对肿瘤细胞的检出率分为86.7%与44.0%，胸腔积液沉淀物的确诊率明显优于细胞学，这和Koksal、Bhanvadia、Jalal等^[5, 6, 15]^的结论一致。Ugurluoglu等^[16]^研究中与细胞涂片相比，沉淀物不仅可以提高MPE的检出率，甚至可增加腺癌、鳞癌等组织病理类型诊断。

通过常规HE染色和免疫组织化学染色，胸腔积液沉淀物诊断的90例MPE患者中有74例明确病理类型，腺癌最常见（67.8%）。需要指出的是，虽然不同来源的转移性腺癌，它们的细胞形态学具有一定的差别^[17]^，但即使经验丰富的病理科医生，仅仅依靠沉淀物的细胞形态学，也很难确定转移性腺癌的原发部位。所以临床医生送检时，应尽可能提供详细的临床资料，尤其是影像学的临床资料，为病理科医生推测原发灶及选择特异性抗体提供依据。

103例患者共送检141次沉淀物标本，多数送检标本由自然静止法获得，标本2 h-6 h内能形成满意的组织块，若静置的时间超过6 h，细胞核可能会出现裂解，加大识别恶性细胞的难度，甚至误诊。本研究中，23例标本起初仅有少量分散的沉淀物或絮状物，不能直接固定，在应用血凝集法后形成了血凝块，最大程度的保证送检机会。

我们的数据显示，绝大多数患者送检1次-2次即可明确诊断，3次以上的送检不能增加阳性率（3.3%），且会增加患者额外的费用。Bielsa等^[18]^报道，第3次送检的标本，仅仅确定了10.3%的病例。根据指南推荐^[19]^，若连续送检两次胸水细胞学结果均为阴性，不推荐再次送检。

终上所述，通过自然静置法或血液凝集法获得的胸腔积液沉淀物具有操作简单、创伤小、花费低、易推广、不需要特殊设备等优点。胸水沉淀物不仅可以明显增加肿瘤细胞的检出率，还可以帮助确定MPE病理类型及原发灶，甚至对于存在有创检查禁忌症或者一般状态较差的患者，胸腔积液沉淀物检查可能是唯一的确诊方法。该项检查的独特诊断优势具有广阔的临床应用前景。
